# In Vitro Evaluation of Desensitizing Agents Containing Bioactive Scaffolds of Nanofibers on Dentin Remineralization

**DOI:** 10.3390/ma14051056

**Published:** 2021-02-24

**Authors:** Natália Bastos-Bitencourt, Marilia Velo, Tatiana Nascimento, Cassiana Scotti, Maria Gardennia da Fonseca, Luiz Goulart, Lucio Castellano, Sergio Ishikiriama, Juliana Bombonatti, Salvatore Sauro

**Affiliations:** 1 Department of Operative Dentistry, Endodontics and Dental Materials, Bauru School of Dentistry, University of Sao Paulo, Bauru, SP 17011-220, Brazil; nembastos@hotmail.com (N.B.-B.); mariliavelo@yahoo.com.br (M.V.); cassikoch@hotmail.com (C.S.); serginho@usp.br (S.I.); julianafraga@usp.br (J.B.); 2 Center for Fuels and Materials (NPE—LACOM), Program of Post-Graduation in Chemistry, Federal University of Paraíba (PPGQ-UFPB), João Pessoa, PB 58033-455, Brazil; tatirln@hotmail.com (T.N.); mgardennia@gmail.com (M.G.d.F.); 3 Laboratory of Nanobiotechnology (NANOS), Institute of Biotechnology, Federal University of Uberlândia, Av. Pará, 1720, Uberlândia, MG 38400-902, Brazil; lrgoulart@ufu.br; 4 Human Immunology Research and Education Group (GEPIH), Technical School of Health, Federal University of Paraíba, João Pessoa, PB 58059-900, Brazil; luciocastellano2@gmail.com; 5 Dental Biomaterials and Minimally Invasive Dentistry, Department of Dentistry, Cardenal Herrera-CEU University, CEU Universities, C/Santiago Ramón y Cajal, s/n., Alfara del Patriarca, 46115 Valencia, Spain; 6 Department of Therapeutic Dentistry, I.M. Sechenov First Moscow State Medical University, 119146 Moscow, Russia

**Keywords:** dentin hypersensitivity, sensitivity, therapy, nanofibers, hydroxyapatites

## Abstract

This study evaluated the effect of the incorporation of bioactive nanofibers in desensitizing agents on dentin permeability. Sixty disks of dentin were randomly distributed in four groups (*n* = 15). Distribution was based on the desensitizing agents, fluoride varnish and self-etching adhesive, and the presence of nanofibers: C (self-etching adhesive Clearfil SE Bond), CN (Clearfil SE Bond with 1% nanofiber), D (Duraphat varnish), and DN (Duraphat varnish with 1% nanofiber). Dentin permeability was determined using hydraulic conductivity. For a qualitative analysis, confocal laser microscopy and scanning electron microscopy were performed. The C group showed the lowest hydraulic conductance (Lp%) (89.33), while the DN group showed the highest Lp% (116.06). No statistical significance was observed in the Lp% values in all groups after the treatment and 6% citric acid challenge (*p* > 0.239). In the images, the CN group presented a higher superficial and intratubular deposition. In addition, this group presented a more homogeneous dentin surface and wide occlusion of dentinal tubules than the other treatments. Despite there being no statistical differences among the treatments employed, the images showed that the CN group presented a higher surface and intratubular deposition compared to the other treatments, even after the acid challenge.

## 1. Introduction

Dentin hypersensitivity (DH) can be classified as a pain symptom arising from exposed dentin, associated or not to noncarious cervical lesions, in response to thermal, mechanical, osmotic, and chemical stimuli [1]. DH is a common clinical condition, with 74% prevalence in the general population [2]. Furthermore, it is likely to be a more frequent dental complaint in the next years owing to the increase in the longevity of dentition [3]. Although pain occurs for a short duration, it is sharp and negatively affects the quality of life of patients [3].

One of the most accepted hypotheses for the mechanism of DH is the hydrodynamic theory reported by Brännström and Aström in 1967 [4]. Under DH, the dentinal tubules are frequently open, leading to an increased dentin permeability. The hydrodynamic theory has shown that even a partial reduction in the functional radii of the dentinal tubules will lead to a reduction in fluid flow, thereby reducing pain symptoms [5].

The management of DH has been focused on analgesia caused by obliteration of dentinal tubules [6], mainly using desensitizing agents [7]. However, the instability of dentinal tubule occlusion and recurrence of hypersensitivity may result in desensitizing agents being a palliative solution for painful symptoms. One of the major drawbacks of the desensitizing agents is the requirement of at least 2–4 weeks to relieve DH symptoms [8]. Therefore, the development of more effective therapies is required considering their long-term performance.

Adhesive systems seem to exhibit a more long-term effect because they create a layer that mechanically blocks the exposed dentinal tubules. These systems include the use of topical fluoride (F) varnishes by precipitating calcium fluoride (CaF_2_) and thus, decreasing dentinal permeability hypersensitivity [9]. F varnishes have shown clinical effectiveness [10]; however, they require multiple applications to take effect because most of the F ions are released within two weeks [11]. This occurs because CaF_2_ precipitates are formed on the outer dentin and could be washed away by saliva or toothbrushing, thereby reopening the dentinal tubules and triggering hypersensitivity. However, although not a definitive treatment, the application of dentin adhesive products can block dentin tubules as a semi-invasive technique in more severe cases [12] because the composites can effectively seal the dentinal tubules by forming a hybrid layer [13].

Advances in nanotechnology have also led to the development of bioactive products for hypersensitivity by introducing compounds that form insoluble precipitates. Silica (SiO_2_)-containing bioactive glasses and calcium phosphate-based bioceramics have been extensively used in the treatment of DH [14]. SiO_2_-based bioactive glasses can nucleate a hydroxyapatite (HAP)-like layer as a product of the chemical reaction between their surface and saliva, thereby occluding the exposed dentinal tubules and reducing the driving of the hydrodynamic stimuli [15]. Bioactive glass particles are shown to be promising in the long-term management of DH; however, in the oral cavity, they can be washed away by the saliva, exposure to an acid challenge, or by toothbrushing [7]. This limits their effects if applied in a coating method. Therefore, novel drug-delivery systems with improved mechanical resistance through an acid challenge would be highly desirable in the field of dental materials.

In tissue engineering, scaffolds of nanofibers are promising biomaterials for regeneration applications because they can impart bioactivity and act as a platform for therapeutic ion release, including different configurations such as 3D scaffolds [16], nanofiber blankets [17], microspheres [18] films/membranes, and foams [19]. Scaffolds of nanofibers have promising characteristics because they combine the desirable properties of the inorganic phase with those of the organic phase and can effectively improve the mechanical properties of dental materials, as shown in a previous study [20]. Moreover, they can induce apatite deposition in a biomimetic delivery system [21].

As calcium phosphate is the main component of dentin, the incorporation of a hybrid nanofiber composed of SiO_2_ and a stable calcium incorporated in the desensitizing agents appears to be promising for the treatment of DH. Furthermore, it even improves the mechanical resistance of the desensitizing agents through an acid challenge or by forming a more insoluble HAP-like layer. In addition, the remineralizing potential of SiO_2_ can be enhanced at nanoscale and also provide great adhesion to calcium phosphate due to its increased surface area relative to volume [22,23,24]. This is a determining factor in HAP formation and might prolong the effect of the desensitizing agent on DH. Hence, the aim of this in vitro study is to evaluate the effect of dentinal tubule occlusion induced by nanofiber scaffolds incorporation (poly lactic acid (PLA) + 30% silica + 4% calcium oxide—CaO) in an adhesive system and F varnish, and to investigate their abilities to resist through a new acid challenge. The null hypothesis was that there are no statistical differences in hydraulic conductance (Lp) among dentine specimens treated with desensitizing agents with or without the addition of nanofibers.

## 2. Materials and Methods

### 2.1. Experimental Design

**Figure 1:**
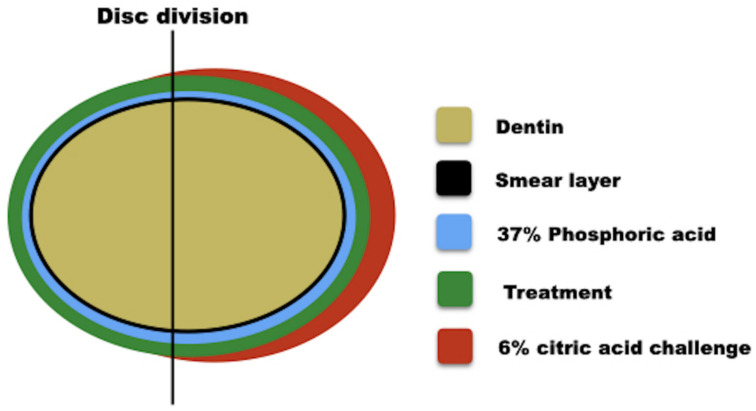
Schematic drawing of the four experimental conditions of the treatment groups.

Sixty dentin discs were obtained from human third molars and were randomized into four groups (*n* = 15): C (self-etching adhesive Clearfil SE Bond-positive control), CN (Clearfil SE Bond with 1% nanofibers), D (Duraphat varnish-positive control), and DN (Duraphat varnish with 1% nanofibers). The response variables were a hydraulic conductivity test, confocal laser scanning microscopy (CLSM), and scanning electron microscopy (SEM). For these variables, the dentin surface was analyzed in the following conditions: (T1) in the presence of the smear layer, (T2) after 37% phosphoric acid etching for 15 s, (T3) after application of the materials tested (Table 1), and (T4) after erosion with 6% citric acid challenge (pH 1.5) for 1 min (Figure 1).

### 2.2. Dentin Sample Preparation

The human third molars of a healthy adult were collected once informed consent was obtained under a protocol approved by the Ethics Committee of Bauru School of Dentistry, University of Sao Paulo (Protocol/90548918.9.0000.5417). Dentin discs were obtained by cutting 4 mm above the cementoenamel junction with a diamond disc (XL-12205, Extec Corporation, Enfield, CT, USA). These discs had an average thickness of 1 ± 0.2 mm and a diameter of 8 ± 0.5 mm. The specimens were selected by acid-etching with 37% phosphoric acid for 15 s. The samples were rinsed and the maximum permeability was evaluated, which was assigned a value of 100% permeability. The dentin discs were randomly divided into four groups (*n* = 10) according to the experimental treatments (Table 1). Before performing the permeability analysis, all the specimens were stored in artificial saliva (pH 7.0) composed of Ca (1.5 mM), PO_4_ (0.9 mM), KCl (150 mM), and Tris buffer (20 mM) containing NaN 0.02% [25].

### 2.3. Nanofibers Fabrication

The nanofiber scaffolds were produced by adapted solution blowing and combined air brushing techniques using a set-up described in previous studies [26,27,28,29]. The hybrid fibers were produced using a PLA obtained from NatureWorks, Jamplast Inc., Ellisville, MO, USA, in pellet form. Precursor solutions of SiO_2_ (30%) and calcium (20% obtained from Gelest PA-USA) were used to develop nanofibers.

### 2.4. Adhesive System and Varnish Samples Preparation

Nanofibers (1 wt.%) were manually incorporated into the adhesive system and the F varnish by mixing for 10 s. Both the materials and nanofibers were weighed on a precision scale (Denver Instrument, São Paulo, Brazil). The percentage of fibers was determined in a previous study [20] to obtain a material with adequate fluidity. For the adhesive system, a tooth primer was deposited for 20 s on the dentin discs and thoroughly dried for 5 s with mild air. A uniform bond film was applied on the dentin discs and light-activated for 10 s using a light-emitting device (Valo, Ultradent) operating at 1000 mW cm^−2^ at a distance of 1 mm. The varnish was applied using microbrushes (KG Brush, KG Sorensen, Cotia, Brazil), and the excess was removed delicately after 4 min with a dental explorer. All the specimens were stored in artificial saliva (pH 7.0) and were maintained in an incubator at 37 °C.

### 2.5. Experimental Conditions

The response variables were the dentin permeability obtained from the hydraulic conductivity test (*n* = 11) and the qualitative analysis (*n* = 4) of the dentin surface images exposed to the treatments under the following conditions: (T1) in the presence of a smear layer created by an abrasive paper #600 (Buehler Ltd., Lake Bluff, IL, USA), to obtain the minimum permeability (PMin); (T2) after 37% phosphoric acid etching for 15 s, to obtain maximum permeability (PMax); (T3) after application of the materials tested (Table 1); and (T4) after erosion with 6% citric acid challenge (pH 1.5) for 1 min (final permeability), to simulate clinical conditions (Figure 1).

### 2.6. Dentin Permeability Analysis

Dentin permeability measurements were performed using a Flodec machine (DeMarco Engineering, Geneva, Switzerland) [30]. Each specimen was connected to Flodec using a plexiglass split-chamber device between two rubber O-rings, providing a surface area standardization of 0.282 mm^2^ under simulated deionized water pulp pressure (20 cm H_2_O). The fluid flow was measured by the movement of an air bubble within a 25 mL capillary glass. Bubble displacement was recorded for 5 min, and the fluid flow was converted to Lp by dividing the fluid flow (μL min^−1^) by the exposed dentin surface area (cm^2^) and the water pressure (cm H_2_O). The permeability of each disc was expressed in percentage as Lp% and tested under the four conditions, i.e., T1, T2, T3, and T4 (Figure 1).

### 2.7. Scanning Electron Microscopy (SEM) Analysis

The morphologies of the dentinal tubules were evaluated after testing under the four conditions (Figure 1). The specimens (*n* = 3) were subjected to critical point drying and were gold coated for SEM (XL30, FEI, Hillsboro, OR, USA) analysis at 10–20 kV [30]. Three photomicrographs were obtained for each condition at 3000× magnification.

### 2.8. Confocal Analysis

Three-point surface images of the dentin discs (*n* = 1) from each group were obtained by confocal microscopy (Leica TCS SPE, Mannheim, Germany) [31] to identify and compare the dentinal tubule obliteration under 40x magnification, at a depth of 20 µm. Analyses were performed after the four conditions were applied (Figure 1), since each disc had an occlusal surface delimited with a #2 spherical carbide drill (KG Sorensen, Sao Paulo, Brazil) in two distinct areas. The materials were applied under the same experimental conditions using hydraulic conductivity. The specimens were divided according to the materials tested, presenting as a control group of discs with a smear layer (minimum permeability) and without a smear layer (maximum permeability). The primary reflection and fluorescence images were overlapped and analyzed on the XY and XZ axes.

### 2.9. Statistical Analysis

Statistical analysis was performed using the software SPSS 16.0 (SPSS Inc., Chicago, IL, USA). Dentin permeability was analyzed by two-way analysis of variance (ANOVA) (using treatment and experimental dentin condition values), followed by Tukey’s multiple comparison tests. The level of significance was set at 5% (*p* < 0.05). Qualitative analyses were performed for SEM and confocal images.

## 3. Results

### 3.1. Dentine Permeability Measurements

Dentin permeability (Lp) results are presented as percentages of maximum permeability (100%) after 37% phosphoric acid etching. Table 2 shows the data from the different treatments under four experimental dentin conditions. The two-way ANOVA test showed a significant effect only for the four experimental dentin conditions (*p* < 0.001).

From the Lp% values listed in Table 2, it was observed that group C presented the lowest Lp% value for the minimum permeability (55.86) compared to that for the maximum permeability (*p* = 0.003). For the CN group, the lowest Lp% value was found at the minimum permeability (47.57) compared to the other conditions (*p* < 0.02). For the DN group, the lowest Lp% value was found at the minimum permeability (59.79) compared to the maximum permeability (100; *p* = 0.008) after the treatment (106.06; *p* = 0.003) and after the 6% citric acid challenge (130.86; *p* = 0.003).

### 3.2. SEM Evaluation

**Figure 2:**
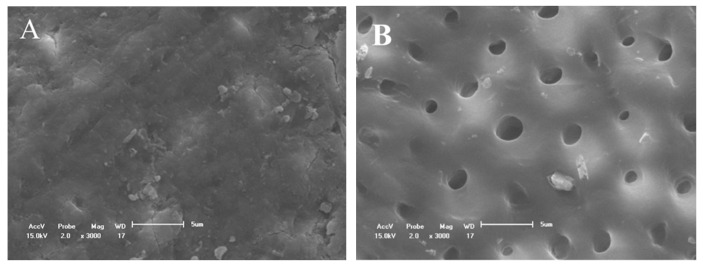
SEM micrographs of the dentine surface morphology at 3000× magnification. (**A**) With smear layer and (**B**) without smear layer. The exposition of dentinal tubules occurred after acid challenge (**B**).

Figure 2A shows the obliteration of the dentinal tubules on the surface before etching with 37% phosphoric acid. After the etching, almost all the dentinal tubules were opened due to the removal of the smear layer. In addition, the peritubular dentin was dense and homogeneous (Figure 2B).

**Figure 3:**
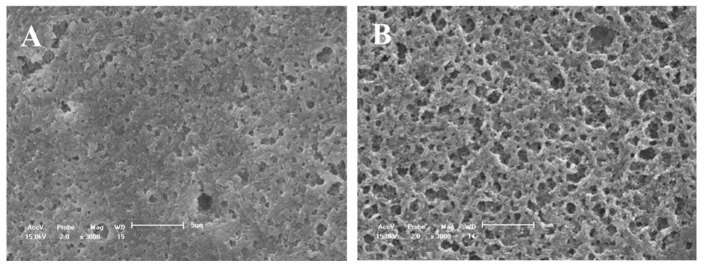
SEM micrographs of the dentine surface morphology at 3000× magnification. (**A**) Adhesive without nanofiber and (**B**) after 6% citric acid challenge. A more porosity surface was verified in (**B**).

**Figure 4:**
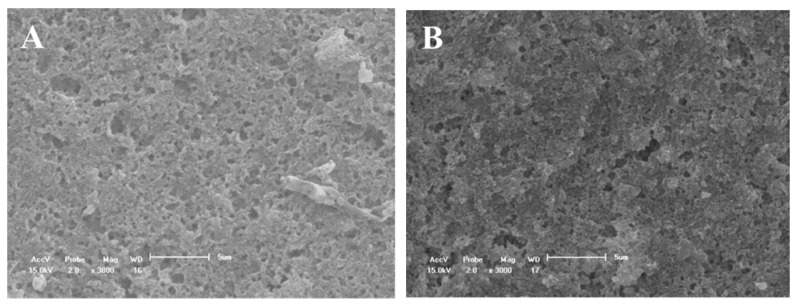
SEM-micrographs of the dentine surface morphology at 3000× magnification. (**A**) adhesive with nanofiber and (**B**) after 6% citric acid challenge. No changes were observed after acid challenge (**B**).

**Figure 5:**
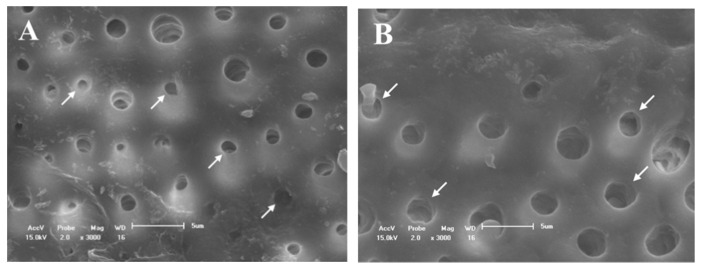
SEM micrographs of the dentine surface morphology at 3000× magnification. (**A**) Fluoride varnish without nanofiber and (**B**) after 6% citric acid challenge. The arrows indicated the partial tubules occlusion with crystal deposition (**A**,**B**).

**Figure 6:**
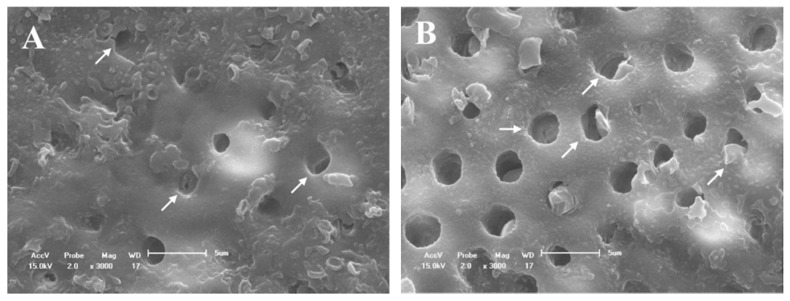
SEM micrographs of the dentine surface morphology at 3000× magnification. (**A**) Fluoride varnish with nanofiber and (**B**) after 6% citric acid challenge. The arrows indicated the regions with crystals adhered to the dentinal tubules (**A**,**B**).

All the evaluated treatments produced morphological changes in the dentin surfaces (Figure 3, Figure 4, Figure 5 and Figure 6). For group C, the SEM images showed a wide occlusion of the dentinal tubules (Figure 3A) before the 1 min of (6%) citric acid challenge. However, after the acid challenge, the dentin surface was more porous (Figure 3B). The CN group showed a more homogeneous and uniform dentin surface with a higher dentinal tubule occlusion as compared to group C (Figure 4A,B) before and after the acid challenge.

For group D, the dentinal tubules were partially open, but with a visually smaller diameter (Figure 5A). After the acid challenge, most deposits were removed; however, the tubules were not completely opened, and some deposits were still adhered to the tubule walls (Figure 5B). On the dentin surfaces of the DN group, crystal deposition was observed at the mouth of the dentinal tubules, thus characterizing a partial occlusion of these dentinal tubules (Figure 6A). However, after the acid challenge, the tubules were partially open with crystals adhered to the wall and mouth (Figure 6B). Therefore, a more obliterated dentin surface was observed for group DN as compared to that for group D.

In general, the CN group had a more homogeneous dentin surface and wide occlusion of dentinal tubules as compared to other treatments.

### 3.3. Confocal Evaluation

**Figure 7:**
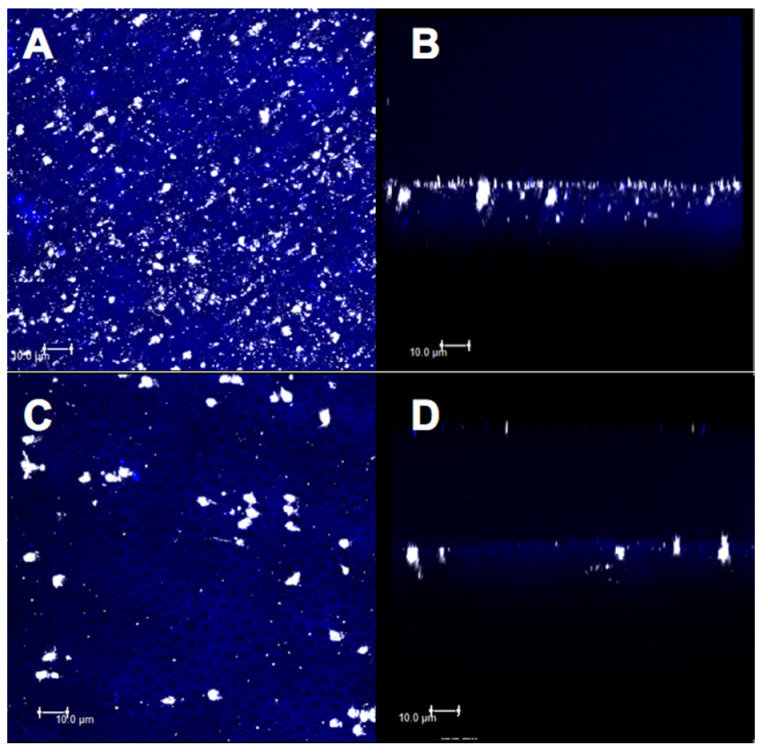
Confocal laser scanning microscopy images (fluorescence mode) in XY and XZ axis of dentin surface. (**A**,**B**) With smear layer and (**C**,**D**) without smear layer.

**Figure 8:**
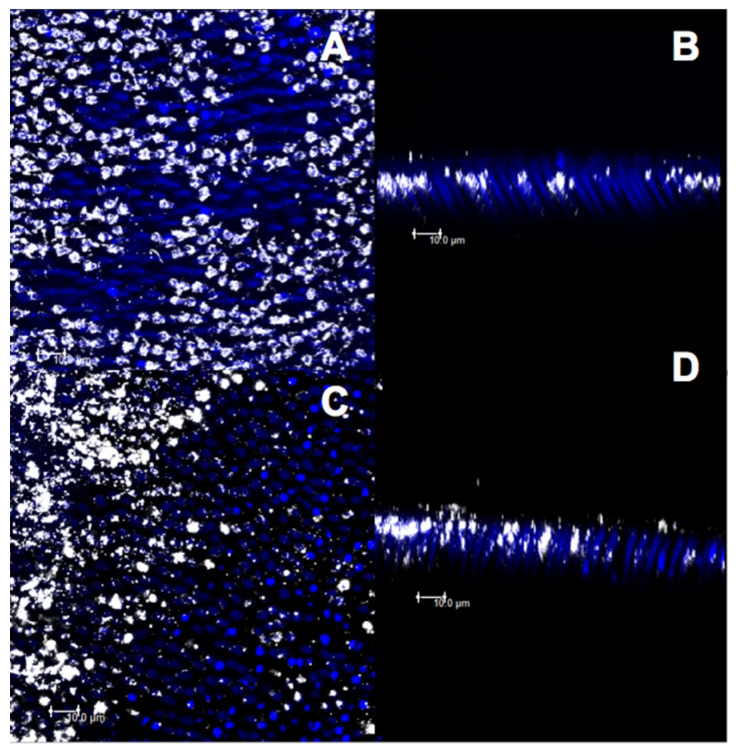
Confocal laser scanning microscopy images (fluorescence mode) in XY and XZ axis of dentin surface. (**A**,**B**) Adhesive without nanofiber and (**C**,**D**) after 6% citric acid challenge.

**Figure 9:**
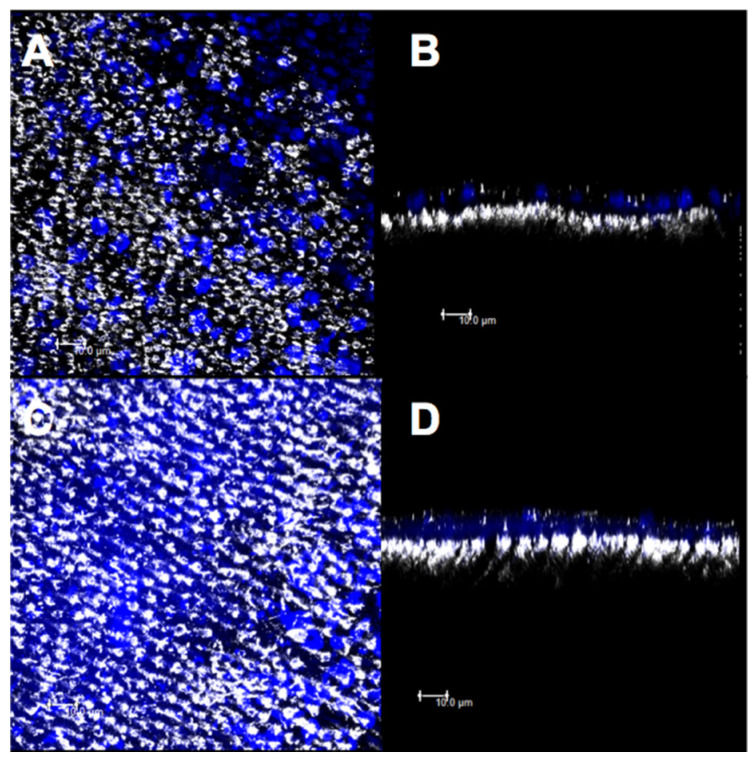
Confocal laser scanning microscopy images (fluorescence mode) in XY and XZ axis of dentin surface. (**A**,**B**) Adhesive with nanofiber and (**C**,**D**) after 6% citric acid challenge.

**Figure 10:**
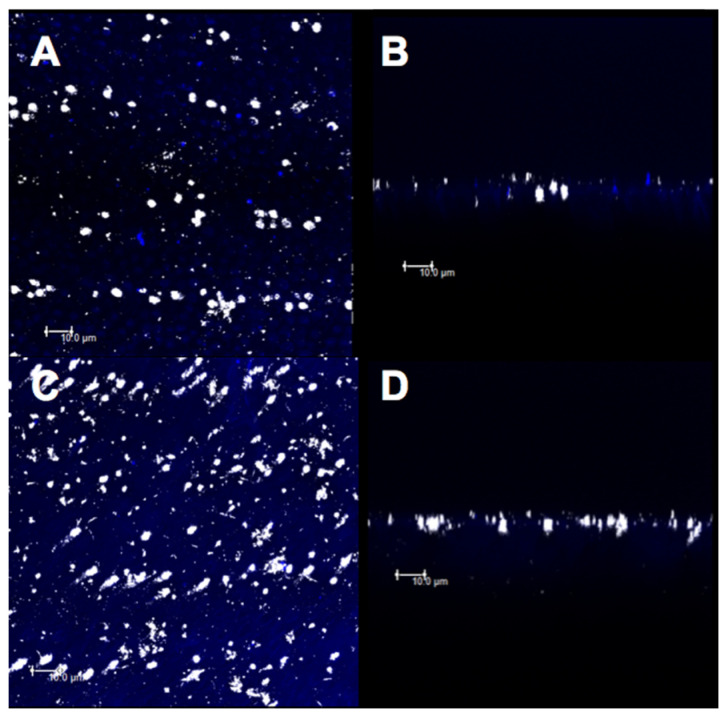
Confocal laser scanning microscopy images (fluorescence mode) in XY and XZ axis of dentin surface. (**A**,**B**) Fluoride varnish without nanofiber and (**C**,**D**) after 6% citric acid challenge.

**Figure 11:**
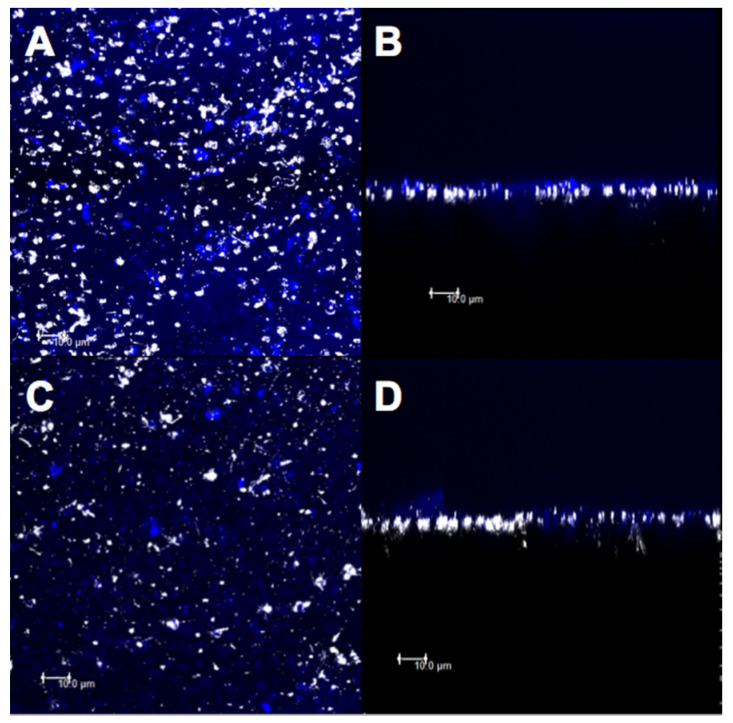
Confocal laser scanning microscopy images (fluorescence mode) in XY and XZ axis of dentin surface. (**A**,**B**) Fluoride varnish with nanofiber and (**C**,**D**) after 6% citric acid challenge.

The micrographs obtained by confocal microscopy were standardized for all the specimens from all the experimental groups (Figure 7, Figure 8, Figure 9, Figure 10 and Figure 11). Bright spots can be seen, corresponding to the obstruction of dentinal tubules by the smear layer debris (Figure 7A,B). After etching with 37% phosphoric acid, the dentinal tubules, which were completely obstructed at the minimum permeability condition (Figure 7B), were partially or completely exposed (Figure 7C,D).

For group C, the deposition of the material on a large part of the superficial dentin (Figure 8A) and large particle concentration at the mouth and inside the dentinal tubules were observed before the citric acid challenge, thus characterizing an intratubular obliteration (Figure 8B). After the acid challenge, less superficial (Figure 8C) and intratubular depositions of the material were observed (Figure 8D).

The images for group CN showed a higher deposition of material on the superficial dentin and a higher obliteration of the dentinal tubules (Figure 9A,B). However, after the acid challenge, a similarity was observed between the images of superficial (Figure 9C) and intratubular (Figure 9D) depositions.

After the treatment with group D and before the citric acid challenge, less deposition of the material on the dentin surface (Figure 10A) and less penetration of the varnish in the dentinal tubules (Figure 10B) were observed. After the acid challenge, the material remained deposited on the surface (Figure 10C) and inside the dentinal tubules (Figure 10D). However, the DN group showed a higher superficial deposition of the material (Figure 11A,C) as well as higher obliteration of dentinal tubules (Figure 11B,D), before and after the acid challenge.

Considering the results of the qualitative images of the different treatments proposed, it was observed that using the adhesive with nanofibers (CN) presented larger superficial and intratubular depositions as compared to that in the other treatments.

## 4. Discussion

One of the main issues of desensitizing agents is the instability of the occlusion [13], thus requiring at least 2–4 weeks to relieve the symptoms of DH [8]. Scaffolds of nanofibers may better spread the material in the dentinal tubules because the nanofibers might act as a carrier of the material into the dentinal tubules and have greater longevity due to the large surface area-to-volume ratio of the nanofibers [32], which could better react with the commercial materials used. Based on the results, the nanofibers could be successfully incorporated into the desensitizing agents because they have a higher potential for dentinal tubule obliteration (Figure 9B,D and Figure 11B,D). Although the present study demonstrated that all the treatments had a similar effect on dentin permeability, the morphological changes in the dentin surfaces were observed in all the groups containing scaffolds of nanofibers. Therefore, the null hypothesis was accepted, since no Lp statistical difference was verified among all groups.

In the current study, we evaluated dental permeability using Flodec [30,33,34,35]. In this method, dentin permeability was measured through the movement of dentinal fluids and calculated by Lp% values [34]. The effectiveness of the desensitizing agents was evaluated when the Lp% values were similar to those obtained in the presence of the smear layer, representing minimum permeability [34]. Our results showed that only group D presented a dentin permeability similar to the minimum permeability, which can be explained by the mechanical barrier created by CaF_2_ precipitation on the dental surface [13,36] and the ability to form a reservoir of CaF_2_ due to high fluoride concentration [37]. In addition, the F varnish may have created a more stable crystallization retention inside the dentinal tubules, as shown in a previous study [34]. This mechanical barrier affects the dentin permeability and DH [38]; however, the short duration of the anti-hyperesthesia effect is still a challenge [39].

The SEM micrographs of the dentin surface showed that the DN group (Figure 6A,B) presented a more obliterated dentin surface than group D (Figure 5A,B), even after the acid challenge. In addition, the DN group demonstrated higher deposition of crystals in the wall and mouth (Figure 5B), although the partial occlusion of the dentinal tubules in group DN (Figure 6B) was similar to that of group D (Figure 5B). The higher obliteration of the dentinal tubules in the group containing the nanofibers can be explained by the formation of an HAP-like layer by the presence of silica, since silica-based bioactive glasses can react with saliva and HAP-like layers [15,40]. Further research using these modified desensitizers is needed to prove if the particles formed represent HAP-like layers and present bioactivity by simulating body fluid analysis.

The topical application of self-etching adhesives has been used to manage DH [41,42]. For better adherence to dentin over dentin desensitizers [13], the present design also evaluated the effect of nanofiber incorporation into self-adhesives. Although the permeabilities of all the groups were similar, the morphological changes were observed on the dentin surfaces for group CN since it (Figure 4A,B) showed a more homogeneous and uniform surface with a higher dentinal tubule occlusion as compared to those of group C (Figure 3A,B), before and after the acid challenge. This can be attributed to the self-etching adhesives producing an acid-resistant hybrid layer [43]. The presence of silica also contributed to the formation of a more homogeneous surface due to the higher intratubular deposition of the material (Figure 9B,D). These results are relevant because the incorporation of these nanofibers could improve the mechanical resistance of the desensitizing agents after new erosion challenges. The enhanced mechanical resistance of these modified materials makes them promising desensitizing agents for long-term action. To the best of our knowledge, only one study has incorporated hybrid nanofibers into dental materials and showed improved mechanical properties of resin-based cement [20], which could strengthen this hypothesis; however, further studies are required to confirm this.

The release of therapeutic ions (Sr^+2^, Ag^+^, Fe^+2^, F^−1^, and Cu^+^) from inorganic–organic hybrid nanofibers encourages the use of nanofibers in the engineering of dental materials [44]. Silica-based bioactive nanofibers have been widely used for biomedical applications [45]. The addition of silica at the nanoscale within the nanofibers used in this study may increase the adherence to calcium phosphate present in dentin due to the increase in the surface area-to-volume ratio [22,24]. Moreover, the formation of an insoluble HAP-like layer is expected. Confocal analysis was effective in evaluating the obliteration of the dentinal tubules because bright spots in dentinal tubules were found that corresponded to either the smear layer or the materials used in this study. Both the desensitizing agents with nanofibers, i.e., CN and DN, showed a higher deposition of the material in the superficial dentin (Figure 8A,C and Figure 11A,C) and a higher obliteration of the dentinal tubules (Figure 8B,D and Figure 11B,D), thus indicating that the nanoscale silica may have facilitated the penetration of the experimental material. However, the CN group demonstrated a higher obliteration as compared to that of the DN group, since the resin matrix of the adhesive system may have shown better intramolecular hydrogen bonds with the nanofibers due to the surface area to volume ratio [32]. This facilitated penetration into the dentinal tubes. In addition, clusters of nanofibers may have been formed, thus making it difficult for the varnish to penetrate the dentin surface.

Although the addition of the nanofibers into desensitizing agents showed no statistical difference in Lp%, more tubular obliterations were found for the nanofiber groups. Such results make nanofibers attractive compounds for use in desensitizing agents for treating dentin hypersensitivity, especially after the acid challenge where the material remained deposited on the surface. Considering that nanofibers have also shown great mechanical properties in the dental materials field [20,29], it could be used as mechanical barrier in further acid challenges in remineralizing products such as dentifrices, varnishes, and adhesives to inhibit erosion development and progression in a long-term performance. However, the choice of desensitizing agents is not important to determine the patient’s predisposing factors [46] to the risk of dentin exposure [47]. The thickness and shape of the specimens and the absence of enamel and pulp horn from the specimens analyzed in this study limit the clinical significance of the findings of this study. In addition, the comparison of different percentages of nanofiber addition into these materials could promote different results. Additionally, dentin permeability measurements require the prior application of 37% phosphoric acid before the application of treatments and this fact does not simulate clinical conditions; however, this limitation can be overcome in future research with the use of other methods for assessing dentin permeability or through clinical research that does not involve this step. To validate the results obtained in this study, further research should be conducted to analyze the clinical performance of scaffold nanofibers in desensitizing agents, so that the symptoms of DH may also be assessed. Additionally, the obliteration action of nanofibers should be further compared to bioactive glass particles as it represents the gold standard in bone regenerative repair and possesses excellent bioactivity [21].

## 5. Conclusions

Based on the results, although all treatments had similar permeabilities, the desensitizing agents with nanofibers were potentially effective as occlusive agents based on qualitative analysis. Further studies to investigate the effect of nanofiber-based desensitization in the clinical scenario are recommended.

## Figures and Tables

**Table 1:** Description of brand name, manufacturer, composition, and procedure of all materials used in this study.

Material	Manufacturer	Composition	Procedure
Clearfil SE Bond	Kuraray, Sakazu, Kurashiki, Okayama, Japan	Primer: Water, MDP, HEMA, hydrophilic dimethacrylates, camphorquinone. Bond: MDP, Bis-GMA, HEMA, camphorquinone hydrophobic dimethacrylate, N/N-diethanol p-toluidine bond, colloidal silica.	Apply primer (20 s), and gently air dry Apply adhesive, gently air dry, light cure (10 s)
Duraphat	Colgate^®^, Sao Bernardo do Campo, SP, Brazil	1 mL of this suspension contains 50 mg sodium fluoride, equivalent to 22.6 mg fluoride, in an alcoholic solution of natural resins.	Passively apply for 4 min

**Table 2:** Mean and standard deviation of the Lp values (%) of the different treatments under four experimental dentin conditions (*n* = 11).

Material	Minimum (With Smear Layer)	Maximum (After Acid Etching)	Treatment	Final (After Erosive Challenge)
Clearfil SE Bond C	55.86 (37.28) Aa	100 Ab	89.33 (40.56) Aab	87.72 (42.80) Aab
Clearfil SE Bond + nanofiber CN	47.57 (41.23) Aa	100 Ab	103.35 (28.19) Ab	106.41 (43.13) Ab
Duraphat varnish D	79.19 (46.60) Aa	100 Aa	106.07 (47.92) Aa	120.61 (65.89) Aa
Duraphat varnish + nanofiberDN	59.79 (27.52) Aa	100 Ab	116.06 (23.87) Ab	130.86 (32.06) Ab

Same uppercase letter in column and lowercase letter in line indicates no difference at the 5% significance level. No statistical significance was observed in the Lp% values in all the groups after the treatment and 6% citric acid challenge (*p* > 0.239).

## Data Availability

Data sharing is not applicable to this article.
